# The HA and NS Genes of Human H5N1 Influenza A Virus Contribute to High Virulence in Ferrets

**DOI:** 10.1371/journal.ppat.1001106

**Published:** 2010-09-16

**Authors:** Hirotaka Imai, Kyoko Shinya, Ryo Takano, Maki Kiso, Yukiko Muramoto, Saori Sakabe, Shin Murakami, Mutsumi Ito, Shinya Yamada, Mai thi Quynh Le, Chairul A. Nidom, Yuko Sakai-Tagawa, Kei Takahashi, Yasuyuki Omori, Takeshi Noda, Masayuki Shimojima, Satoshi Kakugawa, Hideo Goto, Kiyoko Iwatsuki-Horimoto, Taisuke Horimoto, Yoshihiro Kawaoka

**Affiliations:** 1 Division of Virology, Department of Microbiology and Immunology, Institute of Medical Science, The University of Tokyo, Tokyo, Japan; 2 ERATO Infection-induced Host Responses Project, Saitama, Japan; 3 The International Center for Medical Research and Treatment, Kobe University, Kobe, Japan; 4 National Institute of Hygiene and Epidemiology, Hanoi, Vietnam; 5 Avian Influenza Laboratory, Tropical Disease Centre, Airlangga University, Surabaya, Indonesia; 6 Department of Special Pathogens, International Research Center for Infectious Diseases, Institute of Medical Science, The University of Tokyo, Tokyo, Japan; 7 School of Medicine and Public Health, University of Wisconsin-Madison, Madison, Wisconsin, United States of America; Mount Sinai School of Medicine, United States of America

## Abstract

Highly pathogenic H5N1 influenza A viruses have spread across Asia, Europe, and Africa. More than 500 cases of H5N1 virus infection in humans, with a high lethality rate, have been reported. To understand the molecular basis for the high virulence of H5N1 viruses in mammals, we tested the virulence in ferrets of several H5N1 viruses isolated from humans and found A/Vietnam/UT3062/04 (UT3062) to be the most virulent and A/Vietnam/UT3028/03 (UT3028) to be avirulent in this animal model. We then generated a series of reassortant viruses between the two viruses and assessed their virulence in ferrets. All of the viruses that possessed both the UT3062 hemagglutinin (HA) and nonstructural protein (NS) genes were highly virulent. By contrast, all those possessing the UT3028 HA or NS genes were attenuated in ferrets. These results demonstrate that the HA and NS genes are responsible for the difference in virulence in ferrets between the two viruses. Amino acid differences were identified at position 134 of HA, at positions 200 and 205 of NS1, and at positions 47 and 51 of NS2. We found that the residue at position 134 of HA alters the receptor-binding property of the virus, as measured by viral elution from erythrocytes. Further, both of the residues at positions 200 and 205 of NS1 contributed to enhanced type I interferon (IFN) antagonistic activity. These findings further our understanding of the determinants of pathogenicity of H5N1 viruses in mammals.

## Author Summary

Highly pathogenic H5N1 influenza A viruses have caused more than 500 human infections with approximately 60% lethality in 15 countries and continue to pose a pandemic threat. The recent worldwide spread of pandemic H1N1 influenza A viruses raises the concern of reassortment between the H5N1 viruses and other influenza viruses. However, the molecular determinants for high virulence of the H5N1 viruses in mammals are not fully understood. We, therefore, investigated their virulence in a ferret model, which is a widely accepted animal model for assessing human influenza virus replication. We identified an amino acid in hemagglutinin and four amino acids in nonstructural proteins that are associated with high virulence of a human H5N1 virus, A/Vietnam/UT3062/04. We also found that the amino acid in hemagglutinin changes its receptor-binding property and the amino acids in nonstructural protein 1 affect its interferon antagonistic ability. These findings provide insight into the pathogenesis of H5N1 viruses in mammals.

## Introduction

In 1997, the first human case of influenza caused by an H5N1 virus occurred in Hong Kong [1], [2]. In 2003, a new outbreak of H5N1 virus was identified in Vietnam. Since then, H5N1 viruses have spread across Asia, Europe and Africa. As of July 22, 2010, 501 cases of H5N1 virus infections in humans have been reported by the World Health Organization (WHO; http://www.who.int/en/), 297 of which were fatal. The mortality is, therefore, approximately 60%. H5N1 viruses have been characterized by using a variety of mammalian models [3]. In mice, enhanced HA cleavability, as well as lysine at position 627 of the polymerase subunit PB2, plays an important role in the virulence of H5N1 viruses [4]. Viruses possessing these properties replicate systemically and cause death in mice.

Ferrets are considered suitable for evaluating infection of human influenza viruses because these viruses replicate in the upper respiratory tract without adaptation in ferrets, and some strains cause severe pneumonia in these animals. Some of the H5N1 viruses isolated from humans can kill ferrets, whereas H5N1 viruses isolated from birds tend to cause mild disease in this animal model [5], [6]. Systemic infection, high replication efficiencies, and neurovirulence are associated with the high lethality of human H5N1 viruses in ferrets. Salomon et al. [7] reported that the genes encoding the nonstructural proteins (NS) and polymerase complex are important for the lethality of the human H5N1 virus A/Vietnam/1203/04 in ferrets, compared with the avian H5N1 virus A/quail/Vietnam/36/04. However, the molecular bases for the high virulence of H5N1 viruses in ferrets are not fully understood. To advance our understanding of the pathogenicity of H5N1 viruses, we compared the virulence of H5N1 influenza viruses isolated from humans in a ferret model. By generating reassortant viruses between the most virulent A/Vietnam/UT3062/04 (UT3062) virus and the avirulent A/Vietnam/UT3028/03 (UT3028) virus, we identified the genes responsible for high virulence in ferrets. We also performed in vitro studies to determine the molecular mechanisms by which H5N1 viruses exhibit high virulence in mammals.

## Results

### Virulence of Human H5N1 Influenza Viruses in Ferrets

To compare the virulence of H5N1 influenza viruses isolated from humans in ferrets, we intranasally inoculated 5- to 7-month-old male animals (n = 3) with 10^7^ plaque-forming units (PFU) of virus and observed the lethality, changes in body weight and body temperature, clinical signs, and virus shedding in the upper respiratory tract of the virus-infected animals (Table 1). The UT3062, A/Vietnam/UT3040/04, A/Vietnam/UT3028II/03, A/Vietnam/UT30850/05, A/Vietnam/UT3030/03, A/Vietnam/UT3040II/04, and A/Vietnam/UT3047III/04 were virulent in ferrets, causing the deaths of the virus-infected animals. These virulent viruses, with the exception of A/Vietnam/UT3028II/03, caused mean maximum weight loss of 9.1%–18.4% and anorexia, consistent with previous studies [5], [6]. Systemic viral infection was observed in most of the fatally infected animals (Table S1). Notably, inoculation of animals with UT3062 resulted in 100% lethality with 15.4±2.7% mean maximum weight loss (Table 1). These results demonstrate that UT3062 is the most virulent in ferrets of the viruses we tested. By contrast, six other human H5N1 viruses, A/Vietnam/UT30259/04, A/Vietnam/UT3035/03, A/Indonesia/UT3006/05, UT3028, A/Vietnam/UT30408III/05, and A/Vietnam/UT30262III/04 did not kill any ferrets and all, except A/Vietnam/UT30259/04, caused limited body weight loss (1.6%–6.6% mean maximum weight loss, median 4.2%) (Table 1), indicating that H5N1 viruses isolated from humans differ in their virulence in ferrets. Among the H5N1 viruses listed in Table 1, two viruses, A/Vietnam/UT3035/03 and A/Vietnam/UT30408III/05, which did not kill any ferrets, were isolated from patients who recovered from their H5N1 virus infections. The rest of the viruses used were isolated from patients who ultimately died. Of note, the virulence of test viruses in ferrets generally correlated with that in mice [8].

**Table 1:** Virulence of H5N1 influenza viruses in ferrets.

Virus	No. of dead/total	Day of death a	Virus shedding in nasal washes on indicated day p.i. (log_10_) b		Clinical signs			
			3	6	Weight change (%) e	Temperature increase (°C) e	Neurological signs f	Anorexia f
A/Vietnam/UT3062/04	3/3	6,6,8	4.7±0.5	2.1±0.5	−15.4±2.7	2.0±0.4	0/3	3/3
A/Vietnam/UT3040/04	2/3	6,9	4.5±0.2	2.7, - c	−13.6±3.7	2.5±0.1	3/3	3/3
A/Vietnam/UT3028II/03	2/3	4,5	4.2±1.1	- d	−5.8±6.7	2.2±0.6	0/3	1/3
A/Vietnam/UT30850/05	1/3	7	3.6±0.3	2.6±1.8	−18.4±2.1	2.1±0.2	1/3	3/3
A/Vietnam/UT3030/03	1/3	7	4.6±0.3	2.1, 1.5, -	−12.0±5.2	2.5±0.4	2/3	2/3
A/Vietnam/UT3040II/04	1/3	7	4.4±0.5	4.2, -,-	−10.5±4.0	2.8±0.3	1/3	2/3
A/Vietnam/UT3047III/04	1/3	8	3.6±0.3	1.8, 1.0, -	−9.1±3.6	2.3±0.4	1/3	3/3
A/Vietnam/UT30259/04	0/3	-	3.3±2.0	-, -, -	−12.3±6.7	1.7±0.1	0/3	3/3
A/Vietnam/UT3035/03	0/3	-	2.6±0.2	1.8, -,-	−6.6±5.7	2.1±0.3	0/3	0/3
A/Indonesia/UT3006/05	0/3	-	4.7±0.7	-, -, -	−5.2±2.5	2.3±0.3	2/3	1/3
A/Vietnam/UT3028/03	0/3	-	3.8±0.4	-, -, -	−4.8±1.7	2.0±0.5	0/3	2/3
A/Vietnam/UT30408III/05	0/3	-	NA	NA	−3.0±1.4	1.6±0.7	0/3	0/3
A/Vietnam/UT30262III/04	0/3	-	NA	NA	−1.6±0.9	0.7±0.5	0/3	0/3

a Ferrets were infected with 10^7^ PFU of virus in 0.5 ml. Any ferrets exhibiting hemorrhage from a body orifice or the inability to remain upright were euthanized under deep anesthesia.

b Nasal washes were collected from ferrets on days 3 and 6 post-infection and titrated in MDCK cells. Mean titers, expressed as log_10_ PFU/ml with standard deviations, are shown. When virus was not recovered from all ferrets, individual titers were shown. -, titer of <1.0 log_10_ PFU/ml; NA, not available.

c Virus titers of two ferrets, since one of the three ferrets died before day 6.

d Virus titer of one ferret, since two of the three ferrets died before day 6.

e Mean maximum changes in body weight and body temperature with standard deviations.

f No. of animals/total.

On days 3 and 6 post-infection (p.i.), we collected nasal washes from the virus-infected animals and titrated them in Madin-Darby canine kidney (MDCK) cells. On day 6 p.i., the virus titers in the nasal washes of animals infected with the virulent viruses (except for A/Vietnam/UT3028II/03) were generally higher than those of animals infected with viruses that were not lethal (Table 1).

### Generation of UT3062 and UT3028 by Reverse Genetics

**Figure 1:**
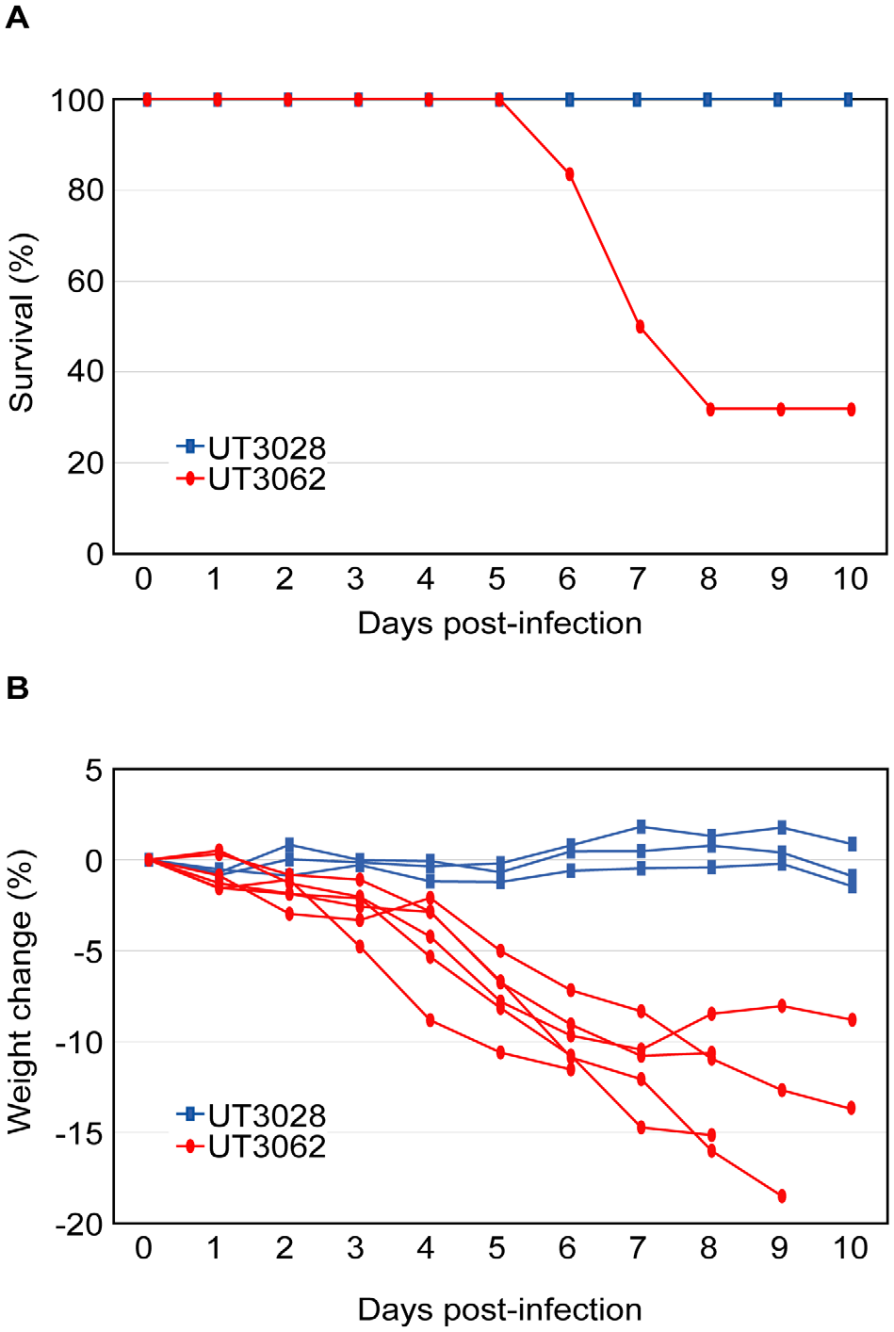
UT3062 differs from UT3028 in its virulence in ferrets. (A) Survival of ferrets infected with 10^7^ PFU of UT3062 (n = 6) and UT3028 (n = 3) is indicated. (B) Percent body weight change of each virus-infected ferret is shown.

**Figure 2:**
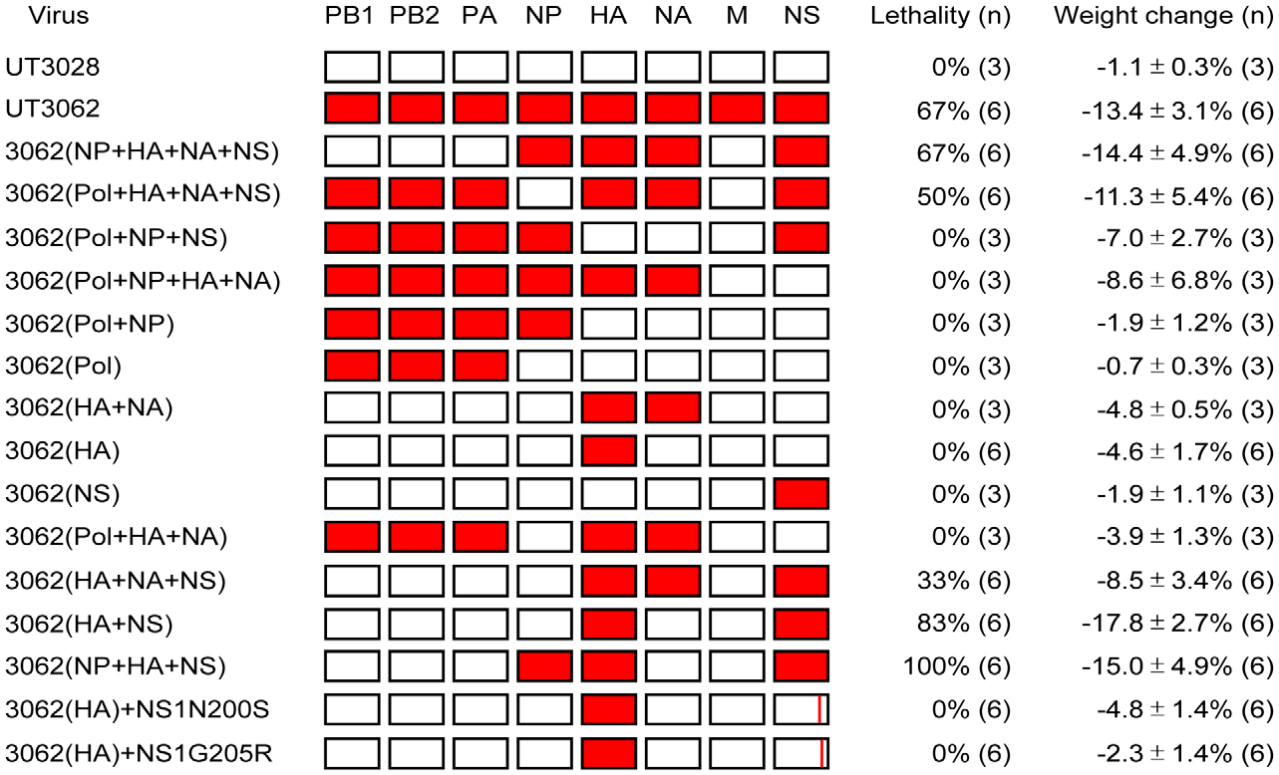
The HA and NS genes contribute to virulence in ferrets. Ferrets were infected with 10^7^ PFU of reassortant viruses that combine the genes of UT3062 (red) and UT3028 (white), generated by reverse genetics, and with the parental UT3062 and UT3028 viruses. Lethality, mean maximum weight change ± standard deviations (SD), and the number of ferrets used (n) are indicated. Animals with greater than 20% weight loss, hemorrhage from any body orifice, or inability to remain upright were euthanized. Pol, polymerase complex. HA+NA, surface glycoproteins, HA and NA.

Sequence comparisons of the viruses used in this study revealed that the most virulent virus UT3062 was most closely related to an avirulent virus UT3028, with 18 amino acid differences in their 9 proteins (Table S2); there were no amino acid differences in the matrix (M) proteins of the two viruses. Therefore, we generated UT3062 and UT3028 by reverse genetics and confirmed their virulence by intranasally inoculating 5- to 6-month-old male ferrets with 10^7^ PFU of the viruses. Animals infected with the UT3062 virus died on days 6–8, resulting in 67% lethality and showed −13.4±3.1% mean maximum weight loss (Figures 1 and 2). Conversely, all animals infected with the UT3028 virus survived and showed appreciably less mean maximum weight loss (−1.1±0.3%) (Figures 1 and 2). These results were similar to those obtained with the respective original viruses (Figure 2 and Table 1).

### Contributions of the HA and NS Genes to the Virulence of UT3062 in Ferrets

To determine the molecular basis for the high virulence of UT3062, we generated reassortant viruses between the UT3062 and UT3028 viruses using reverse genetics and tested their virulence by intranasally inoculating 5- to 6-month-old male ferrets with 10^7^ PFU of the viruses and observing them for 10 days for clinical manifestations. Since no amino acid differences were identified in M proteins of the two viruses, we used the UT3028 M gene for generating all reassortant viruses. The reassortants were named according to the origin of their UT3062 or UT3028 genes. For example, 3062(Pol+NP+HA+NA) indicates a virus possessing the polymerase complex (PB1, PB2, and PA), nucleoprotein (NP), HA, and neuraminidase (NA) genes from UT3062 and the rest of its genes from UT3028 (Figure 2). Reassortant viruses possessing both the UT3062 HA and NS genes, 3062(NP+HA+NA+NS), 3062(Pol+HA+NA+NS), 3062(HA+NA+NS), 3062(HA+NS), and 3062(NP+HA+NS), were lethal to animals (33%–100% lethality). By contrast, those possessing either the HA or NS genes of UT3028 were not lethal to any animals. Mean maximum body weight loss in the animals infected with the former viruses (8.5%–14.4%, median 11.4%) tended to be greater than that in the animals infected with the latter viruses (0.7%–8.6%, median 4.1%). Animals infected with 3062(HA+NS) resulted in 83% lethality and 17.8%±2.7% mean maximum weight loss. These results show that the difference in virulence between UT3062 and UT3028 is mainly attributable to both of the HA and NS genes.

Sequence comparison between UT3062 and UT3028 revealed only one amino acid difference in their HA proteins and four amino acid differences in their NS proteins, two in NS1 and two in NS2 (Table 2). Since NS1 and NS2 mRNAs are produced from the same gene segment, with the NS1 mRNA being unspliced and the NS2 mRNA being spliced, the nucleotide alterations can affect both proteins; i.e., the amino acid differences at positions 200 and 205 of NS1 were coupled to those at positions 47 and 51 of NS2, respectively. Therefore, it was impossible to substitute the amino acids only in the NS1 or the NS2 protein. Here, we generated two reassortant viruses with a mutation in the NS segment by reverse genetics. 3062(HA)+NS1N200S possesses the UT3062 HA gene, a mutant NS segment encoding the UT3028 NS1 protein, which has an asparagine-to-serine substitution at position 200 (and encodes NS2 with a threonine-to-alanine substitution at position 47) and the rest of its genes from UT3028. 3062(HA)+NS1G205R possesses the UT3062 HA gene, a mutant NS segment encoding the UT3028 NS1 protein, which has glycine-to-arginine substitution at position 205 (and encodes NS2 with a methionine-to-isoleucine substitution at position 51) and the rest of its genes from UT3028. We then tested their virulence in ferrets as described above. As shown in Figure 2, both of the reassortant viruses, 3062(HA)+NS1N200S and 3062(HA)+NS1G205R, were not lethal to any animals, with 4.8%±1.4% and 2.3%±1.4 mean maximum weight loss, respectively. These results suggest that all of the amino acids in HA and NS proteins contribute to the virulence in ferrets, although it is unclear whether changes in NS1, NS2, or both affect virulence.

**Table 2:** Amino acid differences between H5N1 influenza UT3028 and UT3062.

	Position	UT3062	UT3028
HA^a^	134	A	T
NS1	200	S	N
	205	R	G
NS2	47	A	T
	51	I	M

a H3 numbering.

In addition, 3062(HA+NA+NS) (33% lethality and 8.5±3.4% mean maximum body weight loss) was attenuated compared to 3062(NP+HA+NA+NS) (67% lethality and 14.4±4.9% mean maximum body weight loss). Further, 3062(NP+HA+NS) and 3062 (HA+NS) killed 100% and 83% of animals, respectively. These results suggest that the UT3062 NP gene may enhance virus virulence in ferrets. These results are consistent with previous findings that virulence of influenza virus is multigenic [7], [9], [10].

### Viral Replication in Ferrets and Cell Culture

To understand the basis for the difference in virulence in ferrets among the viruses, we examined the in vitro and in vivo replication of the parental UT3062, UT3028, and the reassortant viruses. For in vitro testing, we compared their growth kinetics in mink lung epithelial (Mv1Lu) cells by infecting these cells with viruses at a multiplicity of infection (MOI) of 0.001 and monitoring the growth kinetics for 48 h. All of the viruses replicated to more than 10^8^ PFU/ml at 36 or 48 h p.i. and the differences in their viral titers were less than one log PFU/ml at each time point (Figure S1), indicating that there were no substantial differences in their replicative ability in these cells.

To examine viral replication in ferrets, we infected animals with 10^7^ PFU of the parental UT3062, UT3028, and selected reassortant viruses (3062(HA+NS), 3062(NP+HA+NS), and 3062(HA+NA+NS)). Virus titers in nasal and tracheal swabs, and organs were examined. On days 3 and 7 p.i., three animals from each infected group were sacrificed for virus titration. As shown in Table 3, UT3062, 3062(HA+NS), 3062(NP+HA+NS), and 3062(HA+NA+NS) were detected systemically on days 3 and 7 p.i., whereas UT3028 was detected mainly in the upper respiratory tracts of ferrets on day 3, but not 7, p.i. The differences in replicative ability of these viruses in ferrets thus correlate with lethality in this animal model.

**Table 3:** Viral titers in tissues of ferrets infected with H5N1 viruses.

Viruses	Tissues	Days p.i. a					
		3			7		
		Animal No.					
		D3-1	D3-2	D3-3	D7-1	D7-2	D7-3
UT3028	Brain	-	-	2.8	-	-	-
	Olfactory Bulb	-	-	-	-	-	-
	Lung	-	-	4.5	-	-	-
	Hilar lymph node	-	-	-	-	-	-
	Liver	-	-	-	-	-	-
	Kidney	-	-	-	-	-	-
	Spleen	-	-	-	-	-	-
	Small Intestine	-	-	-	-	-	-
	Colon	-	-	-	-	-	-
	Nasal Swab	3.6	4.0	3.3	-	-	-
	Trachea Swab	-	-	-	-	-	-
UT3062	Brain	3.0	-	3.0	4.3	-	6.2
	Olfactory Bulb	3.3	4.8	5.3	5.5	-	6.2
	Lung	6.1	5.3	4.0	5.6	-	8.6
	Hilar lymph node	4.5	3.5	3.6	3.5	-	-
	Liver	8.2	6.1	3.7	5.1	-	5.5
	Kidney	4.3	2.0	-	-	-	6.7
	Spleen	4.8	3.6	3.5	3.3	-	7.1
	Small Intestine	3.2	2.3	-	3.7	-	-
	Colon	3.0	-	-	3.7	-	5.5
	Nasal Swab	6.0	3.7	4.8	-	-	4.4
	Trachea Swab	2.6	1.0	3.6	-	-	6.1
3062(HA+NA+NS)	Brain	3.2	-	3.1	5.4	5.7	4.6
	Olfactory Bulb	4.3	5.0	5.4	5.5	5.4	3.3
	Lung	4.6	-	4.5	-	7.3	5.2
	Hilar lymph node	4.3	-	-	-	3.9	2.3
	Liver	5.9	-	5.1	5.2	7.3	5.2
	Kidney	-	-	-	-	4.6	-
	Spleen	4.2	3.4	3.3	-	4.6	-
	Small Intestine	3.3	-	-	-	4.2	2.0
	Colon	-	-	-	2.0	4.0	-
	Nasal Swab	4.5	5.4	4.3	-	5.1	-
	Trachea Swab	3.8	2.5	1.7	2.2	5.1	2.5
3062(HA+NS)	Brain	5.4	5.7	4.0	9.4	6.4	8.4
	Olfactory Bulb	6.6	6.9	6.8	8.1	9.4	9.6
	Lung	7.8	5.8	3.4	7.4	6.5	7.2
	Hilar lymph node	6.5	3.2	2.7	3.5	5.4	5.4
	Liver	8.4	2.8	5.1	6.3	6.3	5.4
	Kidney	5.8	-	-	3.8	2.3	-
	Spleen	6.8	4.6	7.6	5.9	5.4	-
	Small Intestine	4.1	-	2.8	5.0	3.4	3.7
	Colon	4.5	2.3	-	5.7	2.9	-
	Nasal Swab	5.7	4.5	5.0	3.5	3.0	3.2
	Trachea Swab	4.5	2.5	4.0	4.9	3.1	4.2
3062(NP+HA+NS)	Brain	3.5	4.0	5.8	5.8	8.2	6.7
	Olfactory Bulb	4.0	5.7	7.7	5.6	9.4	4.5
	Lung	5.9	5.7	6.5	7.6	7.0	7.4
	Hilar lymph node	4.3	3.5	4.4	6.3	4.0	3.3
	Liver	6.4	6.6	4.9	8.2	6.3	6.2
	Kidney	3.5	3.3	-	6.7	2.0	2.3
	Spleen	4.2	4.7	2.3	6.9	3.3	2.0
	Small Intestine	-	3.1	4.1	6.8	3.4	-
	Colon	-	3.1	3.2	6.6	2.5	-
	Nasal Swab	4.1	5.4	5.6	5.7	3.9	5.2
	Trachea Swab	3.8	2.5	5.0	5.5	3.8	3.2

a Tissues were collected from ferrets infected with 10^7^ PFU of virus on days 3 and 7 pi. The samples were titrated in MDCK cells. The virus titer was calculated as PFU per gram (for tissues) or ml (swabs) and expressed as log_10_ PFU/gram or ml. -, titer of <2.0 log_10_ PFU/g or 1.0 log_10_ PFU/ml.

### Tissue Pathology in Ferrets

**Figure 3:**
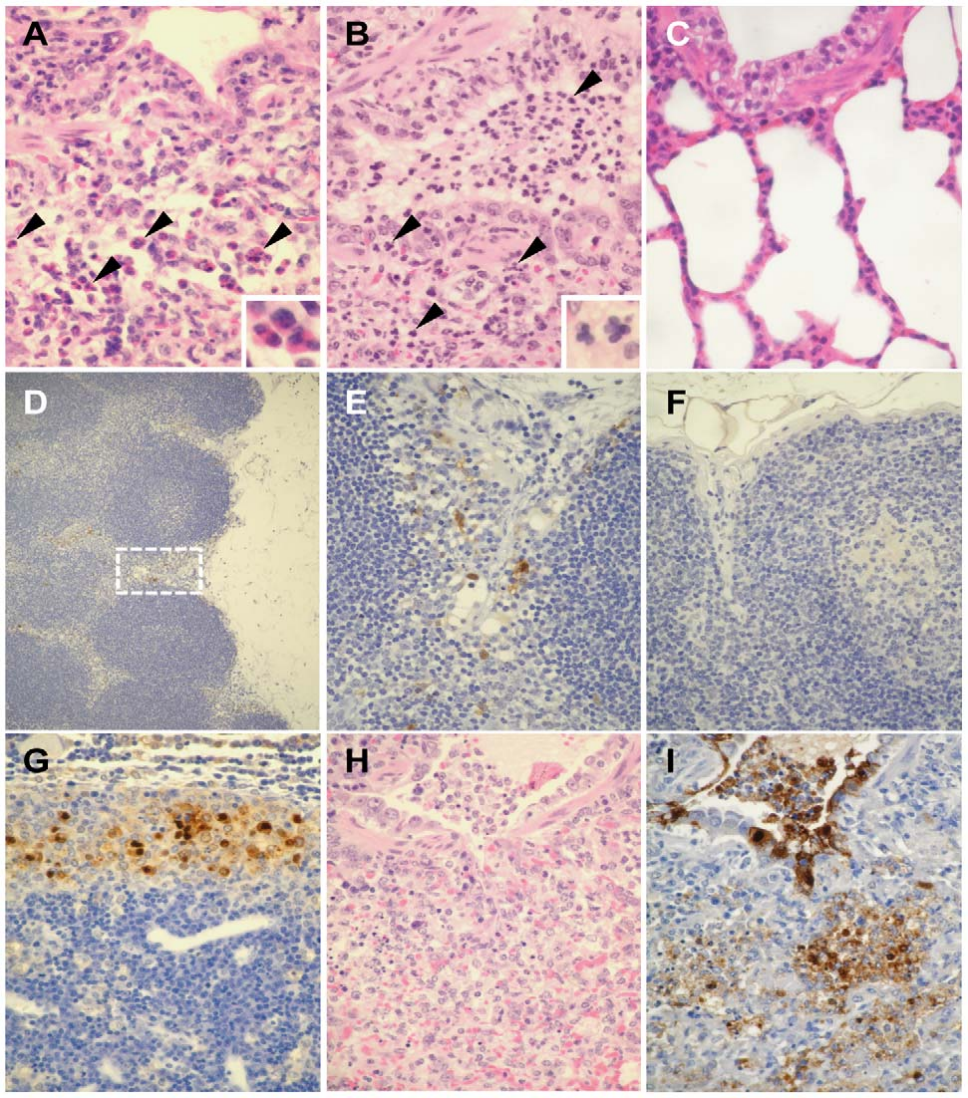
Pathological findings in ferrets infected with UT3062 or 3062(HA+NS). (A) Lungs of ferret infected with UT3062. Infiltration of eosinophils (arrowheads and insert) was obvious around the bronchi 1 day p.i. (B) Lungs of ferrets infected with UT3028. Neutrophils (arrowheads and insert) were apparent in the lung lesion 1 day p.i. (C) The bronchial area of normal appearance has clear air spaces. (D) Immunostaining of tracheobronchial lymph node in a ferret infected with UT3062. Virus antigen can be seen in the sinusoid area (brown pigment) 1 day p.i. (E) Higher magnification of Figure 3D's broken-line square area. Reticular cell-like cells in the sinusoid stain positive for virus antigen (brown pigment). (F) The lymph node with normal appearance did not contain brown pigments. (G) Immunostaining of tracheobronchial lymph node in a ferret infected with 3062(HA+NS). Many viral antigen-positive cells are visible in the sinusoid (brown pigment) 7 days p.i. (H) Lung lesion in a ferret infected with 3062(HA+NS). Prominent inflammatory infiltrates and hemorrhage are detected 7 days p.i. by H&E staining. (I) Immunostaining of Figure 3H lesion. Heavy staining of virus antigen can be seen in the bronchial epithelium and alveolar cells (brown pigments) 7 days p.i.

**Figure 4:**
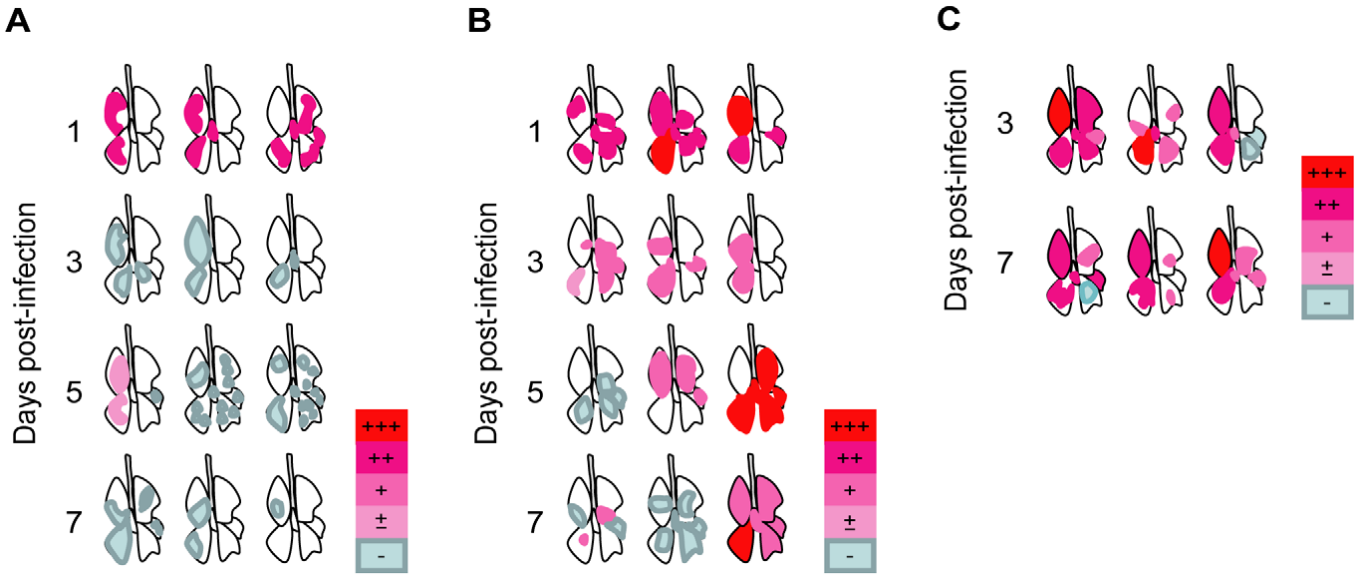
Distribution of lung lesions and viral antigens in ferrets. (A) Lungs of ferrets infected with UT3028. 1, 3, 5, and 7 days p.i. (B) Lungs of ferrets infected with UT3062. 1, 3, 5, and 7 days p.i. (C) Lungs of ferrets infected with 3062(HA+NS). 3 and 7 days p.i. Viral antigens in lung lesions, +++; many, ++; moderate, +; few, ±; scant, −; not detected. Distributions of lung lesions and viral antigens were confirmed by microscopic examination.

When we compared the histopathology between ferrets infected with UT3062 and those infected with UT3028, we found three major differences (Figures 3 and 4): (1) host reaction to viral exposure in the lungs on day 1 p.i., (2) viral infection in the tracheobronchial lymph node, and (3) distribution of viral antigens and the inflammatory reaction in the lungs on day 3 and beyond p.i. Firstly, cells infiltrating the lung lesions differed between animals infected with UT3062 and those infected with UT3028. Although substantial numbers of viral antigen-positive cells were detected in the lungs of ferrets infected UT3062 or UT3028, the lungs of ferrets infected with UT3062 had marked infiltration of eosinophils around/in the bronchi (Figure 3A). By contrast, the lung lesions of ferrets infected with UT3028 contained many neutrophils (Figure 3B). Secondly, viral infection in the tracheobronchial (pulmonary regional) lymph node at 1 day p.i. differed between the two viruses. Although we did not detect viral antigen in the tracheobronchial lymph node of ferrets infected with UT3028, we did find viral antigen at this site in all three ferrets infected with UT3062 (Figure 3D and E). Thirdly, in animals infected with UT3028, we did not detect viral antigen beyond 3 days p.i., with the exception of one ferret, which was euthanized at 5 days p.i. (Figure 4A). The numerous neutrophils observed on 1 day p.i. were replaced by lymphocytes, macrophages and regenerative epithelial cells during the course of infection (data not shown). On the other hand, in animals infected with UT3062, a substantial number of viral antigen-positive cells were detected in the lungs even 3 days p.i. and the areas in the lungs where the viral antigen-positive cells were detected expanded widely by 5 and 7 days p.i. in some ferrets (Figure 4B). Moreover, when compared to ferret lung lesions with less viral antigen-positive cells, the lesions of ferrets with extensive viral antigen-positive cells had fewer lymphocytes and substantial pulmonary edema, hemorrhaging and fibrinous exudates (data not shown). These findings indicate that there was a tendency for delay in viral clearance in UT3062-infected ferrets and consequently some animals progressed to death. The virus was, however, completely eliminated in some animals, presumably because of individual animal variability.

Next, to evaluate the effect of the UT3062 HA and NS genes in vivo, we examined the pathogenicity of 3062(HA+NS) virus, which possesses UT3062 HA and NS genes and its remaining genes from UT3028. When we examined ferrets infected with 3062(HA+NS) on days 3 and 7 p.i., we found that they had pathological lesions that more closely resembled those of ferrets infected with UT3062 than those of ferrets infected with UT3028. Namely, the ferrets had viral infection in the tracheobronchial lymph node and widely distributed viral pneumonia by 3 days p.i. (Figure 3G to I). Pulmonary edema, hemorrhages and fibrinous exudates were obvious in the lung lesions rather than recruitment of lymphocytes and regenerative changes, which were characteristic of ferrets infected with UT3028.

Therefore, the UT3062 HA and NS gene products play a critical role in viral pathogenicity in this ferret model. Viruses first replicated in the lungs (at the primary site of viral exposure), and infection then expanded into the tracheobronchial lymph node. Viral infection in the regional lymph node may negatively affect viral exclusion from the host, leading to continued viral replication.

### Characterization of Reassortant Viruses with Mutations in Their Glycoproteins

**Figure 5:**
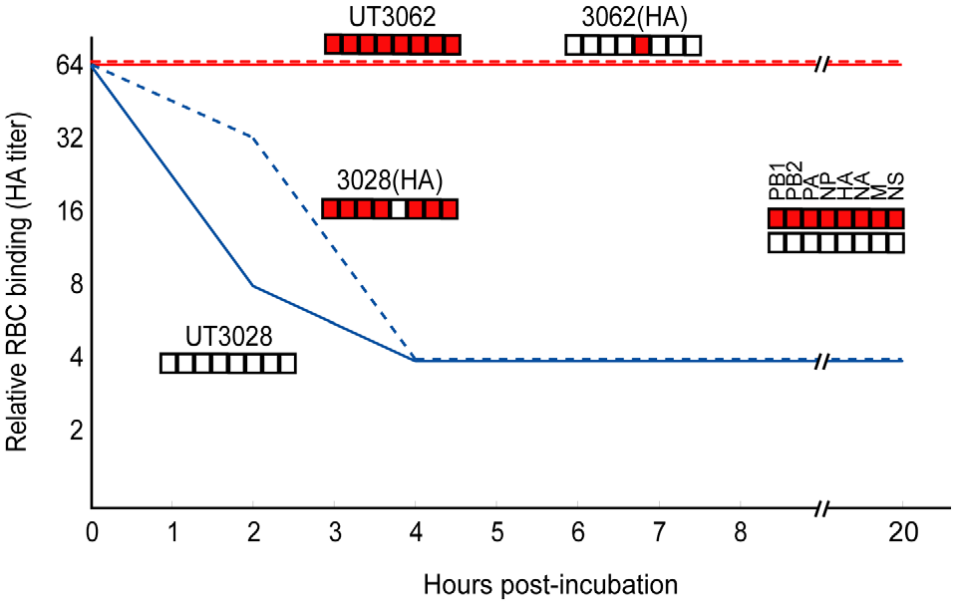
UT3062 and UT3028 differ in their virus elution ability from erythrocyte. Two-fold dilutions of virus containing HA titers of 1∶64 were incubated with equal volumes of 0.55% chicken erythrocytes at 4°C for 1 h. They were then incubated at 37°C and the reduction in HA titers was recorded for 20 h.

UT3062, like almost all other human H5N1 viruses, has alanine at position 134 of HA (H3 numbering). UT3028, however, has threonine at this position (Table 2). These findings suggest that a single substitution at position 134 (A134T) of HA affects virulence in ferrets. Previously, Auewarakul et al. [11] showed that substitutions at positions 129 and 134 (L129V/A134V) allow virus recognition of both sialic acid liked to galactose by α2,3 linkage (SAα2,3Gal) and SAα2,6Gal, unlike the parent virus, which recognizes only SAα2,3Gal. Yamada et al. [12], however, found that a single substitution at position 134 (A134T) did not change receptor-binding preference with the same sialylglycopolymers used by Auewarakul et al. [11]. We, therefore, performed virus elution assays using chicken and horse erythrocytes. From chicken erythrocytes, which express both SAα2,3Gal and SAα2,6Gal [13], UT3062 and a reassortant possessing the UT3062 HA were not eluted even after 20h of incubation at 37°C. By contrast, UT3028 and a reassortant possessing the UT3028 HA were gradually released from chicken erythrocytes (Figure 5). Since the viruses possessing the UT3028 HA were eluted regardless of the origin of the NAs (either UT3028 or UT3062), this difference in elution from erythrocytes is due to the difference in the amino acid residue at position 134 of HA. When we used horse erythrocytes, all of the viruses were more rapidly eluted from these erythrocytes than from chicken erythrocytes; however, UT3028 and a reassortant possessing the UT3028 HA were eluted more efficiently from horse erythrocytes than were UT3062 and reassortants possessing the UT3062 HA (data not shown). These results suggest that UT3062 HA differs from UT3028 HA in its receptor-binding property.

### Interferon Antagonistic Property of Reassortant Viruses with Mutations in NS Gene

NS1 mediates type I IFN antagonism and affects viral growth in cells. We, therefore, assessed the IFN antagonistic activity of these NS1s by using an IFN bioassay [14], [15], [16], [17], [18], [19], [20]. Briefly, Mv1Lu cells were infected with each virus at an MOI of 1.25 and the supernatants were collected at 12–24 h p.i. H5N1 viruses in the supernatants were inactivated with UV and neutralizing antibody (A1A1, [21]) treatment. The supernatants were added to fresh Mv1Lu cells and cultured for 22 h, followed by infection with vesicular stomatitis virus (VSV) to determine VSV infectivity of the above-described supernatant-pretreated Mv1Lu cells. As a control, we used a recombinant influenza virus expressing an RNA-binding- and IFN-antagonism-defective NS1 protein within which two basic amino acids were substituted to alanines (R38A/K41A) on the UT3062 backbone [17], [22]. We also generated reassortant viruses possessing the mutant NS1 of UT3028 that has either the N200S or the G205R mutation, on the UT3028 backbone and designated them 3028NS1-N200S and 3028NS1-G205R, respectively. As described above, these viruses also possessed amino acid substitution in NS2, T47A, or M51I, respectively.

**Figure 6:**
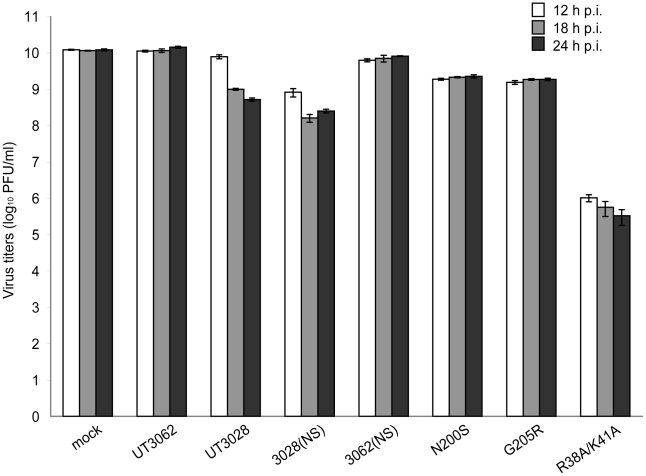
The 3062 NS gene promotes IFN antagonism. Mv1Lu cells were infected with each virus at an MOI of 1.25. Supernatants from virus-infected cells were harvested 12–24 h p.i. After inactivation of viral infectivity, the supernatants were added to fresh Mv1Lu cells and incubated for 22 h. The pretreated Mv1Lu cells were then infected with VSV and infectivity was assessed by plaque assays. N200S represents the virus expressing mutant NS1 protein in which asparagine was substituted to serine and mutant NS2 protein in which threonine was substituted to alanine on the UT3028 backbone. G205R represents the virus expressing mutant NS1 protein in which glycine was substituted to arginine and mutant NS2 in which methionine was substituted to isoleucine on the UT3028 backbone. As a control, we used a recombinant influenza virus expressing an RNA-binding- and IFN antagonism-defective NS1 protein within which two basic amino acids were substituted to alanines (R38A/K41A) on the UT3062 backbone. The data are shown as mean VSV titers (log_10_ PFU/ml) ± SD of triplicate values obtained in a single experiment, and are representative of two independent experiments.

At 18 h and 24 h p.i., the supernatant from Mv1Lu cells infected with UT3028 or a virus possessing the UT3028 NS gene and the remaining genes from UT3062 (i.e., 3028(NS)) inhibited VSV plaque formation more efficiently than did the supernatants from cells infected with UT3062 (statistically significant difference at *P*<0.05, Tukey Honestly Significant Difference [HSD] test) (Figure 6). Furthermore, the supernatant of cells infected with either 3028NS1-N200S or 3028NS1-G205R inhibited VSV plaque formation more efficiently than did that from viruses possessing the UT3062 NS gene (i.e., UT3062 and 3062(NS)) (statistically significant difference, *P*<0.05, Tukey HSD test), but less efficiently than that from viruses possessing the UT3028 NS gene (i.e., UT3028 and 3028(NS)) (Figure 6). These results indicate that both serine at position 200 and arginine at position 205 of NS1 contribute to the enhanced type I IFN antagonistic property of UT3062 NS1, which, in turn, leads to high virulence in ferrets.

To further assess the IFN antagonistic property of NS1, we investigated the effects of the amino acids in NS1 on the expression of the firefly luciferase reporter gene under the control of an interferon-stimulated response element (ISRE) in 293 cells treated with IFNβ. Briefly, 293 cells were transfected with pISRE-Luc, pRL-TK, and pCAGGS NS1 or pCAGGS GFP (negative control). At 24 h post-transfection, the cells were treated with recombinant human IFNβ. At 30 h post-transfection, the cells were lysed and luciferase activities were measured by using the Dual-luciferase Reporter assay system. There were, however, no significant differences in expression from the ISRE between UT3062 NS1 and UT3028 NS1 (data not shown). We then investigated the effects of the amino acids in NS1 on the expression of the firefly luciferase reporter gene under the control of the IFNβ promoter in 293 cells treated with Sendai virus (SeV) as described previously [23]. Briefly, 293 cells were transfected with p125-Luc, pRL-TK, and pCAGGS NS1 or pCAGGS GFP (negative control). At 36 h post-transfection, the cells were treated with SeV (Cantell strain). At 48 h post-transfection, the cells were lysed and luciferase activities were measured by using the Dual-luciferase Reporter assay system. The results of this experiment also showed that there were no significant differences in expression from the IFNβ promoter between UT3062 NS1 and UT3028 NS1 (data not shown), indicating that other mechanisms affect the IFN antagonistic property.

## Discussion

Here, using H5N1 viruses isolated from humans, we found that receptor-binding property and NS1 IFN antagonism play important roles in the high virulence of these viruses in ferrets.

HA is a receptor-binding and fusion protein and, therefore, is required for virus entry. It is known to play a critical role in virulence [4], [24], [25], [26], [27]. In this study, we found that viruses possessing threonine at position 134 of HA were appreciably attenuated in ferrets compared to those possessing alanine. Although Yamada et al. [12] did not find differences in the receptor-binding preference between HAs with a single substitution at position 134 (134A or 134T) in a direct binding assay to sialylglycopolymers, we found that this substitution affected receptor-binding property as detected by a virus elution assay (Figure 5). Since the amino acid at position 134 is located near the receptor-binding pocket but does not directly interact with sialyloligosaccharides [11], the substitution at this residue may influence the receptor-binding property indirectly. Alanine at position 134 of HA is highly conserved in avian H5N1 viruses—only one virus is known to harbor serine at that position (the Influenza Sequence Database (ISD; https://flu.lanl.gov/, registration system [28])). Similarly, most human H5N1 viruses also have alanine at this position; however, UT3028 and two other H5N1 viruses that we isolated from humans have threonine at this position (Y. Sakai-Tagawa and Y. Kawaoka, unpublished). Further, eleven H5N1 viruses isolated from humans have valine and one has serine at this position (ISD; https://flu.lanl.gov/ [28] and Y. Sakai-Tagawa and Y. Kawaoka, unpublished). These data indicate that an amino acid substitution at position 134 of HA is more frequently observed in human H5N1 viruses than in avian viruses, suggesting that viruses possessing a substitution at position 134 of HA may be selected during replication in humans.

Although NS1 is a multifunctional protein, one of its main functions is to suppress type I IFN production [29]. Recent studies revealed that NS1 plays an important role(s) in antiviral responses via dsRNA-dependent protein kinase R (PKR) and 2′5′-oligoadenylate synthetase/RNase L [30], [31]. Here, using an IFN bioassay, we showed that both serine at position 200 and arginine at position 205 of NS1 contribute to the enhanced type I IFN antagonistic property of UT3062 that leads to high virulence in ferrets. However, we did not observe significant differences in IFNβ-stimulated expression from the ISRE or in SeV-stimulated expression from the IFNβ promoter in 293 cells between the UT3062 and UT3028 NS1 proteins. It may be that NS1 exhibits type I IFN antagonism by a mechanism other than tested in this study. Alternatively, the difference observed in the highly sensitive IFN bioassay using VSV is not detectable in other IFN assays. The amino acid residues at positions 200 and 205 of NS1 are not well conserved, although the residues at these positions in UT3062 have been observed in other human and avian H5N1 viruses (Table S3). These findings support the hypothesis that the amino acid residues determined to be important in this study are affected by the genetic background of the test viruses. Nonetheless, the HA amino acid at position 134 and the NS1 amino acids at positions 200 and 205 may now be included as virulence markers for H5N1 viruses.

## Materials and Methods

### Ethics Statement

Our research protocol for the use of ferrets followed the University of Tokyo's Regulations for Animal Care and Use, which was approved by the Animal Experiment Committee of the Institute of Medical Science, the University of Tokyo (approval number: 19–29). The committee acknowledged and accepted both the legal and ethical responsibility for the animals, as specified in the Fundamental Guidelines for Proper Conduct of Animal Experiment and Related Activities in Academic Research Institutions under the jurisdiction of the Ministry of Education, Culture, Sports, Science and Technology, 2006.

### Cells

Madin-Darby canine kidney (MDCK) cells were maintained in minimal essential medium (MEM) with 5% newborn calf serum. Human embryonic kidney 293 and 293T cells were maintained in Dulbecco's modified Eagle's MEM (DMEM) with 10% fetal calf serum. Mink lung epithelial (Mv1Lu) cells were maintained in MEM with 10% fetal calf serum and 1% non-essential amino acids. All cells were grown at 37°C in 5% CO_2_.

### Viruses

H5N1 viruses isolated from humans in Vietnam and Indonesia were used in this study (Table 1). Virus stocks were propagated through two passages in MDCK cells for 24–48 h at 37°C. The cell supernatants were harvested, clarified by centrifugation, aliquoted, and stored at −80°C. The frozen virus stocks were thawed and titrated for virus infectivity in MDCK cells by plaque assays. Virus titers were calculated as PFU/ml. All experiments were performed under biosafety level 3+ conditions.

### Plasmid-Based Reverse Genetics

Viral RNA was extracted directly from the supernatants of H5N1 virus-infected MDCK cell cultures with a QIAamp Viral RNA Mini Kit (Qiagen, http://www1.qiagen.com/). Complementary DNA was generated by SuperscriptIII (Invitrogen, http://www.invitrogen.com/) with the universal primers for influenza A virus genes. The resulting products were PCR-amplified using PfuUltra High-Fidelity DNA polymerase (STRATAGENE, http://www.stratagene.com/) with specific primers for each virus gene and cloned into a plasmid under the control of the human RNA polymerase I (PolI) promoter and the mouse RNA PolI terminator (PolI plasmids). We altered the NS1 gene sequence that encodes the RNA-binding site of UT3062 to create the RNA-binding defective sequence (R38A/K41A) as previously described [17].

All reassortant viruses and the parental UT3062 and UT3028 viruses were generated by plasmid-based reverse genetics, as described by Neumann et al. [32]. Briefly, PolI plasmids and protein expression plasmids were mixed with a transfection reagent, TransIT 293T (Mirus Bio, http://www.mirusbio.com/); incubated at room temperature for 15 min; and then added to 293T cells. Transfected cells were incubated in OPTI-MEM I (Invitrogen) for 48 h. Supernatants containing infectious viruses were harvested and propagated in MDCK cells at 37°C for 48 h. The supernatants were harvested, aliquoted, and stored at −80°C.

### Ferret Study

We used male ferrets, 5–7 months old (MarshallBioResources, http://www.marshallbioresources.com/) in this study. All ferrets were inoculated intranasally with 10^7^ PFU of infectious virus in 500 µl of phosphate-buffered saline (PBS) under anesthesia with ketamine (25 mg/kg) and xylazine (2 mg/kg). Clinical signs, body weights, and body temperatures were recorded daily for 10 days post-infections (p.i.). The percent changes in body weights were calculated by comparing the weights of each ferret at each time point to its initial weight on day 0. Body temperatures were measured using a rectal thermometer. Changes in body temperature were calculated by comparing the body temperatures of each ferret at each time point to its initial body temperature on day 0. All animals exhibiting more than 20% weight loss, hemorrhage from any body orifice, or inability to remain upright were euthanized. Surviving ferrets were euthanized under deep anesthesia at 3 weeks p.i. On days 3 and 6 p.i., nasal washes were collected from anesthetized ferrets and titrated for virus infectivity in MDCK cells by plaque assays.

Ferrets infected with the parental UT3062 and UT3028 and selected reassortant viruses (Table 3) were euthanized with deep anesthesia and necropsied on days 3 and 7 p.i. Tissue samples of the brain, olfactory bulb, lungs, hilar lymph node, liver, kidney, spleen, duodenum, and descending colon were collected. A portion of each was stored at −80°C for virus titration and the rest were preserved in 10% neutral buffered-formalin for pathological examination. To prepare 10% tissue emulsions, frozen tissue samples were thawed, weighed, and homogenized in 10 volumes (w/v) of sterile PBS using a multi-beads shocker (Yasui Kikai, http://www.yasuikikai.co.jp/). After centrifugation of the samples at 800× g for 5 min at 4°C, the supernatants were collected. In addition, swabs from nose and trachea were collected and suspended in 1 ml of sterile PBS containing 0.3% BSA and penicillin (200 U/ml). After centrifugation at 800× g for 5 min at 4°C, the supernatants were collected and stored at −80°C. Virus in nasal washes, swabs and tissue samples was titrated for virus infectivity in MDCK cells by plaque assays. Virus titer was expressed as PFU/g for tissue samples and PFU/ml for nasal washes and swabs. The limitations of virus detection were 10^2.0^ PFU/g for tissue samples and 10^1.0^ PFU/ml for nasal washes and swabs. The formalin-fixed tissues were processed for routine paraffin embedding. The paraffin-embedded tissues were cut into 5 µm thick slices and stained using hematoxylin-and-eosin (H&E). Additional sections were cut for immunohistological staining with rabbit polyclonal antibodies against an H5N1 virus (A/Vietnam/1203/04). Specific antigen-antibody reactions were visualized by means of 3,3′ diaminobenzidine tetrahydrochloride and the Dako EnVision system (Dako, http://www.dako.jp/). All animal experiments were approved by the Animal Research Committee of The University of Tokyo.

### Viral Growth Kinetics in Mv1Lu Cells

The reassortant viruses and the parental UT3062 and UT3028 viruses were inoculated into Mv1Lu cell monolayers at an MOI of 0.001 PFU with MEM containing 0.3% bovine serum albumin, and incubated at 37°C. Cell supernatants were harvested at a given number of hours p.i. After centrifugation at 1,000× g for 5 min, samples were titrated for virus infectivity in Mv1Lu cells by plaque assays.

### Virus Elution Assay

The ability of viruses to be eluted from erythrocytes was assessed as previously described [33], [34], with some modifications. Briefly, virus stocks were diluted serially in calcium saline (0.9 mM CaCl_2_-154 mM NaCl in 20 mM borate buffer, pH 7.2) and 50 µl aliquots were incubated with 50 µl of 0.55% chicken erythrocytes at 4°C for 1 h in microtiter plates. The plates were then transferred to 37°C and monitored periodically for 20 h.

### IFN Bioassay

Levels of IFN secreted by virus-infected Mv1Lu cells were assessed as previously described [14], [15], [16], [17], [18], [19], [20], with some modifications. Briefly, Mv1Lu cells were infected with each virus at an MOI of 1.25. Supernatants from infected cells were harvested 12–24 h p.i. To inactivate viral infectivity, the supernatants were treated with UV light for 20 min and then mixed with neutralizing α-VN1203HA monoclonal antibodies (A1A1, [21]). The supernatants were added to fresh Mv1Lu cells and incubated for 22 h. These pretreated Mv1Lu cells were then infected with VSV, and the VSV infectivity titers were determined. As a control, we used a recombinant influenza virus expressing an RNA-binding- and IFN antagonism-defective NS1 protein within which two basic amino acids were substituted to alanines (R38A/K41A). The experiments were carried out in triplicate and were independently repeated twice.

### IFN Reporter Assay

8×10^4^ of 293 cells were transfected with 50 ng of pISRE-Luc (Clontech, http://www.clontech.com/) and 50 ng of pRL-TK (Promega, http://www.promega.com/) by using TransIT293 (Mirus, http://www.mirusbio.com/). 100 ng of pCAGGS NS1 or pCAGGS GFP were co-transfected. Cells were incubated for 24 h and were treated with 100 units of recombinant human IFNβ, 1a (PBL interferonSource, http://www.interferonsource.com/). At 30 h post-transfection, cells were lysed and luciferase activities were measured by using the Dual-luciferase Reporter assay system (Promega, http://www.promega.com/) according to the protocol provided by the manufacturer. Firefly luciferase values were divided by Renilla luciferase values to normalize for transfection efficiency.

2×10^5^ of 293 cells were transfected with 250 ng of p125-Luc (the reporter plasmid carrying the firefly luciferase gene under the control of the IFNβ promoter was kindly provided by Takashi Fujita) and 250 ng of pRL-TK (Promega, http://www.promega.com/) by using TransIT293 (Mirus, http://www.mirusbio.com/). 5–500 ng of pCAGGS NS1 or pCAGGS GFP were co-transfected. After incubation for 24 h, cells were stripped with trypsin and divided into two wells of a 12-well plate. At 36 h post-transfection, cells were treated with approximately 5×10^10^ focus-forming unit of SeV (Cantell strain). At 48 h post-transfection, cells were lysed and luciferase activities were measured by using the Dual-luciferase Reporter assay system (Promega, http://www.promega.com/) according to the protocol provided by the manufacturer. Firefly luciferase values were divided by Renilla luciferase values to normalize for transfection efficiency.

## Supporting Information

Figure S1 Viral growth kinetics in Mv1Lu cells. The reassortant viruses and the parental UT3062 and UT3028 viruses were infected into Mv1Lu cell at an MOI of 0.001 PFU. The viruses in the cell supernatants were harvested at a given number of hours p.i. and titrated in Mv1Lu cells. Values are the means ± SD of three independent experiments. No appreciable differences were observed in their replication properties in Mv1lu cells.
(0.21 MB TIF)

Table S1 Virus replication in the respiratory tract and other tissues of ferrets fatally infected with H5N1 virus.
(0.04 MB DOC)

Table S2 Amino acid differences between UT3028 and UT3062.
(0.05 MB DOC)

Table S3 Amino acids at positions 200 and 205 of NS1 in human and avian H5N1 viruses.
(0.04 MB DOC)
