# Supplementary figures and images for: The HA and NS Genes of Human H5N1 Influenza A Virus Contribute to High Virulence in Ferrets
Source: PLoS Pathog. 2010 Sep 16;6(9):e1001106. doi: 10.1371/journal.ppat.1001106 (PMC2940759; doi:10.1371/journal.ppat.1001106)

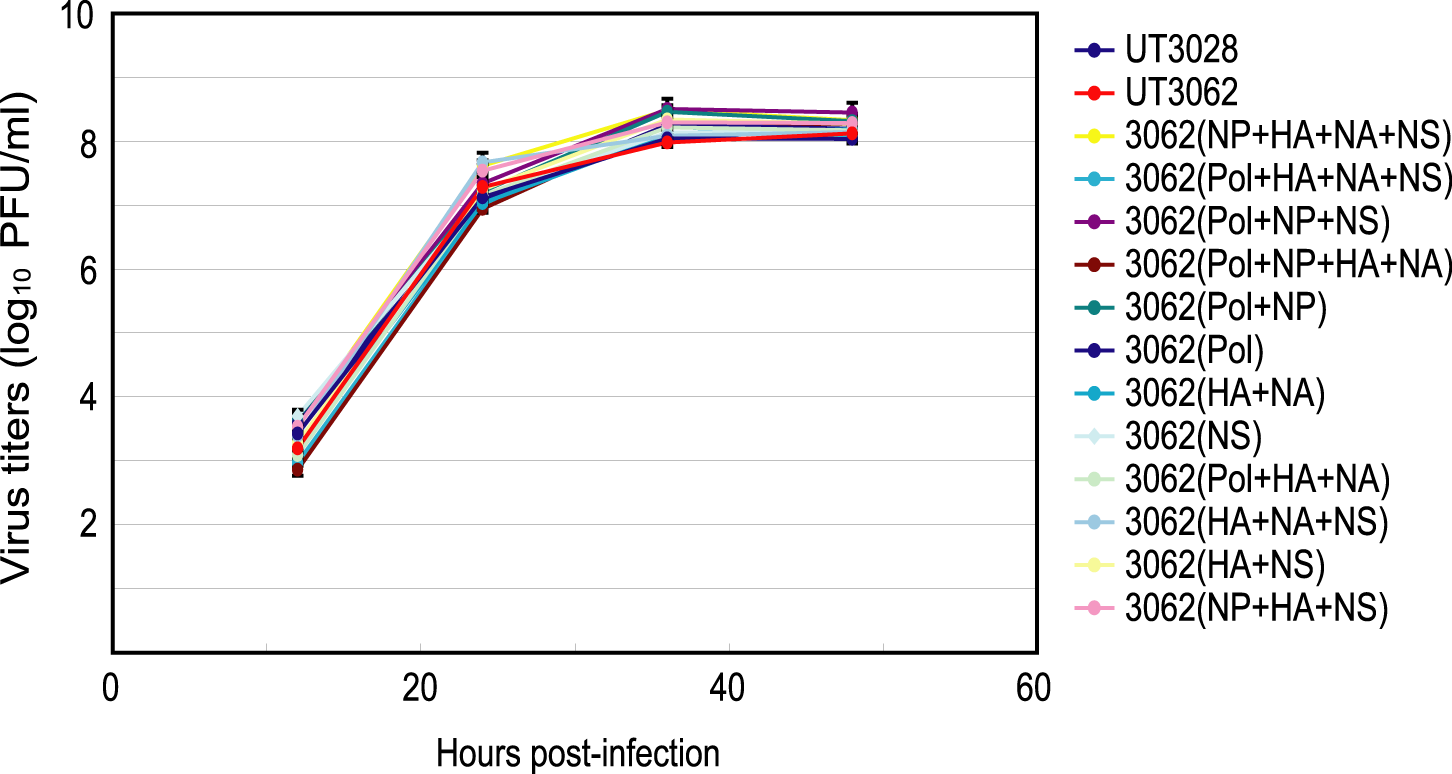

Supplement: Figure S1 — Viral growth kinetics in Mv1Lu cells. The reassortant viruses and the parental UT3062 and UT3028 viruses were infected into Mv1Lu cell at an MOI of 0.001 PFU. The viruses in the cell supernatants were harvested at a given number of hours p.i. and titrated in Mv1Lu cells. Values are the means ± SD of three independent experiments. No appreciable differences were observed in their replication properties in Mv1lu cells. (0.21 MB TIF) [file ppat.1001106.s001.tif]
