# Supplementary material for: The HA and NS Genes of Human H5N1 Influenza A Virus Contribute to High Virulence in Ferrets
Source: PLoS Pathog. 2010 Sep 16;6(9):e1001106. doi: 10.1371/journal.ppat.1001106 (PMC2940759; doi:10.1371/journal.ppat.1001106)
Supplement: Table S1 — Virus replication in the respiratory tract and other tissues of ferrets fatally infected with H5N1 virus. (0.04 MB DOC) [file ppat.1001106.s002.doc]

**Supporting Information**

**Table S1.** Virus replication in the respiratory tract and other tissues of ferrets fatally infected with H5N1 virus

| Viruses ^a^ | Day p.i. | Virus titer (log_10_ PFU/g) | | | |
| --- | --- | --- | --- | --- | --- |
|  |  | Brain | Lung | Spleen | Colon |
| A/Vietnam/UT3062/04 | 6 | 5.5 | 5.5 | 3.0 | 4.3 |
|  | 6 | - | 4.1 | - | 2.5 |
|  | 8 | 2.6 | 7.5 | - | 2.0 |
| A/Vietnam/UT3028II/03 | 4 | - | 3.4 | 2.5 | 2.7 |
|  | 5 | 4.5 | 5.0 | 6.0 | - |
| A/Vietnam/UT3040/04 | 6 | 4.6 | 7.3 | - | 3.2 |
|  | 9 | 4.4 | - | - | - |
| A/Vietnam/UT3030/03 | 7 | 2.8 | 3.3 | 1.0 | - |
| A/Vietnam/UT3040II/04 | 7 | - | 6.6 | 3.3 | 4.6 |
| A/Vietnam/UT30850/05 | 7 | 5.4 | 6.2 | 3.0 | 3.3 |
| A/Vietnam/UT3047III/04 | 8 | 4.0 | 2.3 | - | - |

^a^ Ferrets were intranasally inoculated with 10^7^ PFU of each H5N1 virus. Tissues were collected from the dead ferrets on the indicated days and titers were determined in MDCK cells. -, titer of <2.0 log_10_ PFU/g; p.i., post-infection.
