# Supplementary material for: The HA and NS Genes of Human H5N1 Influenza A Virus Contribute to High Virulence in Ferrets
Source: PLoS Pathog. 2010 Sep 16;6(9):e1001106. doi: 10.1371/journal.ppat.1001106 (PMC2940759; doi:10.1371/journal.ppat.1001106)
Supplement: Table S2 — Amino acid differences between UT3028 and UT3062. (0.05 MB DOC) [file ppat.1001106.s003.doc]

**Table S2.** Amino acid differences between UT3028 and UT3062

|  | Position | UT3062 | UT3028 |
| --- | --- | --- | --- |
| PB2 | 666 | A | T |
|  |  |  |  |
| PB1 | 178 | E | D |
|  | 213 | T | N |
|  | 384 | L | V |
|  | 655 | V | M |
|  |  |  |  |
| PB1-F2 | 89 | T | I |
|  |  |  |  |
| PA | 448 | S | A |
|  |  |  |  |
| NP | 99 | K | R |
|  |  |  |  |
| HA^a^ | 134 | A | T |
|  |  |  |  |
| NA^b^ | 50 | A | S |
|  | 78 | F | L |
|  | 325 | T | N |
|  | 347 | Y | G |
|  | 455 | S | G |
|  |  |  |  |
| NS1 | 200 | S | N |
|  | 205 | R | G |
|  |  |  |  |
| NS2 | 47 | A | T |
|  | 51 | I | M |

^a^ H3 numbering.

^b^ N2 numbering.
