# Supplementary material for: The HA and NS Genes of Human H5N1 Influenza A Virus Contribute to High Virulence in Ferrets
Source: PLoS Pathog. 2010 Sep 16;6(9):e1001106. doi: 10.1371/journal.ppat.1001106 (PMC2940759; doi:10.1371/journal.ppat.1001106)
Supplement: Table S3 — Amino acids at positions 200 and 205 of NS1 in human and avian H5N1 viruses. (0.04 MB DOC) [file ppat.1001106.s004.doc]

**Table S3.** Amino acids at positions 200 and 205 of NS1 in human and avian H5N1 viruses

|  | Position | UT3062 | UT3028 | Human H5N1 viruses ^a^ | Avian H5N1 viruses ^c^ |
| --- | --- | --- | --- | --- | --- |
| NS1 | 200 | S | N | G (86) ^b^ | S (1353) |
|  |  |  |  | S (67) | G (74) |
|  |  |  |  | N (5) | I (29) |
|  |  |  |  | R (2) | N (11) |
|  |  |  |  |  | V (3) |
|  |  |  |  |  | ? (1) |
|  | 205 | R | G | G (156) | G (1416) |
|  |  |  |  | R (4) | R (53) |
|  |  |  |  |  | W (2) |

^a^ Out of 160 human H5N1 influenza viruses in the Influenza Sequence Database, from which redundantly registered ones were excluded, the numbers of viruses possessing the indicated amino acid at each position are shown in parentheses.

^b^ All of the viruses possessing glycine at position 200 of NS1 were isolated in Indonesia.

^c^ Out of 1471 avian H5N1 influenza viruses in the Influenza Sequence Database, from which redundantly registered ones were excluded, the numbers of viruses possessing the indicated amino acid at each position are shown in parentheses.
